# Optimisation by Design of Experiment of Benzimidazol-2-One Synthesis under Flow Conditions

**DOI:** 10.3390/molecules24132447

**Published:** 2019-07-03

**Authors:** Serena Mostarda, Tugçe Gür Maz, Alessandro Piccinno, Bruno Cerra, Erden Banoglu

**Affiliations:** 1 Department of Pharmaceutical Sciences, University of Perugia, Via del Liceo 1, 06123 Perugia, Italy; 2 Current affiliation: Novartis Pharma AG, CH-4002 Basel, Switzerland; 3 Department of Pharmaceutical Chemistry, Faculty of Pharmacy, Gazi University, Etiler, 06560 Ankara, Turkey

**Keywords:** flow chemistry, statistical experimental design, benzimidazol-2-one

## Abstract

A novel flow-based approach for the preparation of benzimidazol-2-one (**1**) scaffold by the 1,1′-carbonyldiimidazole (CDI)-promoted cyclocarbonylation of *o*-phenylenediamine (**2**) is reported. Starting from a preliminary batch screening, the model reaction was successfully translated under flow conditions and optimised by means of design of experiment (DoE). The method allowed the efficient preparation of this privileged scaffold and to set up a general protocol for the multigram-scale preparation in high yield, purity, and productivity, and was successfully applied for the multigram flow synthesis of *N*-(2-chlorobenzyl)-5-cyano-benzimidazol-2-one, which is a key synthon for hit-to-lead explorations in our anti-inflammatory drug discovery program.

## 1. Introduction

**Figure 1:**
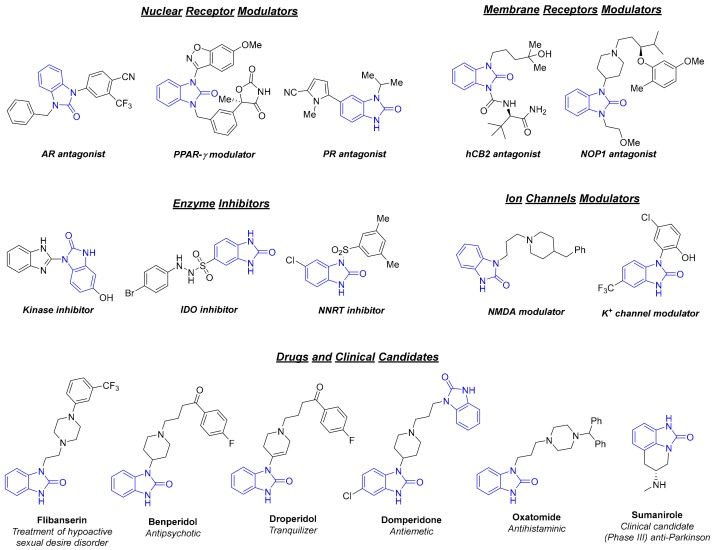
Examples of benzimidazolone-based biologically active derivatives, drugs, and clinical candidates.

With the emergence of high-throughput screening technologies, the development of efficient, practical, and cost-effective synthetic procedures of certain privileged structures, which can be used for compound library design, has become a challenging task for medicinal chemists and drug developers. Diverse nitrogen-containing heterocyclic systems are of crucial importance in medicinal chemistry, as they can be used to expand the drug-like chemical space, thereby increasing the efficiency of drug discovery programs. Among them, benzimidazol-2-one (**1**) is a privileged azaheterocyclic scaffold in medicinal chemistry, being the structural framework of numerous biologically active molecules, pharmaceutically relevant chemical tools, and drugs (Figure 1) [1]. Indeed, benzimidazolone derivatives have recently taken a great deal of attention in the scientific community because they exhibit a plethora of biological activities including antibacterial [2], antifungal [3], antiviral [4], antidiabetic [5], analgesic [6], and anticancer activity [7]. Benzimidazol-2-one (**1**) bears an aromatic backbone fused with a cyclic urea, and is a pharmacophoric motif that is able to interact—by both hydrogen bonding and π stacking—with different biological macromolecules, including nuclear and membrane receptors, enzymes, and ion channels [1,8] (Figure 1). Furthermore, benzimidazolone-containing compounds, such as flibanserin [9], benperidol [10], droperidol [11], domperidone [12], and oxamide [13], represent the active pharmaceutical ingredients (APIs) of well-known blockbuster drugs, while sumanirole [14], a benzimidazolone-fused tricyclic derivative, has been recently reported as a highly potent and selective D2 receptor full agonist currently in phase III clinical trial for the treatment of Parkinson’s disease (Figure 1). Finally, benzimidazolones have also been widely used for the preparation of pigments and polymers in material science [15,16].

Although unsubstituted benzimidazol-2-one (**1**) was synthesised for the first time by Rudolph in 1879 [17], there has been an upsurge of interest in the field during the last 20 years, and considerable attention has been paid to the development of new methods as well as to the improvement of the traditional synthetic protocols for the preparation of benzimidazol-2-one core (**1**) and its derivatives [1,8,18,19]. Traditional synthetic approaches include the cyclocarbonylation of *o*-phenylenediamine (**2**), transformation of benzimidazolium salts, synthesis from arylureas, Curtius reaction of anthranilic acids or phthalic anhydrides, decarbonylative ring contraction of benzodiazepinones or quinoxalinediones, decarbonylative cycloaddition of isocyanates to isatins, and assembly of 2-iodoarylcarbodiimides with acrylates, one-pot reaction of hydroxylamines, aldehydes, and trimethylsilyl cyanide [1,8,18,19]. It is worth noting that a four-step continuous flow synthesis of flibanserin that involved a 1,8-diazabicyclo [5.4.0]undec-7-ene (DBU)-promoted thermal cyclisation of an *N*-Boc-substituted *o*-phenylendiamine derivative as the key step to prepare the benzimidazolone ring has been recently reported [20]. Although a number of synthetic alternatives is currently available, the cyclocarbonylation of *o*-phenylenediamine (**2**) with phosgene, triphosgene, carbon dioxide, carbon monoxide, dimethyl carbonate, urea, and 1,1′-carbonyldiimidazole (CDI) still remains the most widely used approach because of its broad substrate scope and low cost. However, high-boiling organic solvents under harsh conditions, long reaction time, time-consuming extra purification steps, and difficult scalability represent drawbacks of conventional batch processes. In this regard, as widely demonstrated by seminal reports in the field, the high reproducibility of thermal reactions under flow conditions, which is a crucial factor for scale-up operations, is mainly related to the better heat transfer efficiency due to the higher surface-to-volume ratio over conventional batch mode [21]. This allows for rapid, homogeneous, safe, and scale-independent heating that avoids the generation of “hot-spots” and gradients of temperature, which are typical of round-bottom flasks [21].

Following our interest in benzimidazole-based drug discovery [22,23,24,25,26] and in the application of flow technology in medicinal chemistry programs [27,28,29,30], in this paper, we report a novel flow synthesis of benzimidazol-2-one (**1**) by the CDI-promoted cyclocarbonylation of *o*-phenylenediamine (**2**). In particular, with the aim to set up a general method for enabling an efficient multigram-scale preparation of key synthons for hit-to-lead explorations, we applied the following workflow: (i) batch screening of the model reaction, (ii) translation under flow conditions and optimisation by statistical design of experiment (DoE) [31], and (iii) application of the optimised protocol for the gram-scale flow synthesis of *N*-(2-chlorobenzyl)-5-cyano-benzimidazol-2-one (**3**), which will be used in our medicinal chemistry efforts towards anti-inflammatory drug discovery targeting 5-lipoxygenase-activating protein (FLAP) [26,32].

## 2. Results and Discussion

### 2.1. Translation under Flow Conditions and Optimisation by Design of Experiment (DoE)

With the aim to define suitable conditions for translating the reaction under flow modality, 1,1′-carbonyldiimidazole (CDI) was selected as a safe and eco-friendly carbonylating agent [33,34] while polyethylene glycol (PEG) 300, in mixture 3:7 (*v*/*v*) with tetrahydrofuran (THF), was added as the co-solvent because of its eco-friendly profile [35] and the ability to favor the solubilisation of all the reaction components. Thus, the reaction was performed under batch modality by adding a 0.3 M solution of CDI (2.2 equiv.) in THF/PEG300 (7:3, *v*/*v*) to a 1 M solution of **2** in THF/PEG300 (7:3, *v*/*v*) and the resulting solution was refluxed (160 °C) for 16 h, affording the desired benzimidazol-2-one (**1**) in 40% isolated yield after chromatographic purification. 

**Figure 2:**
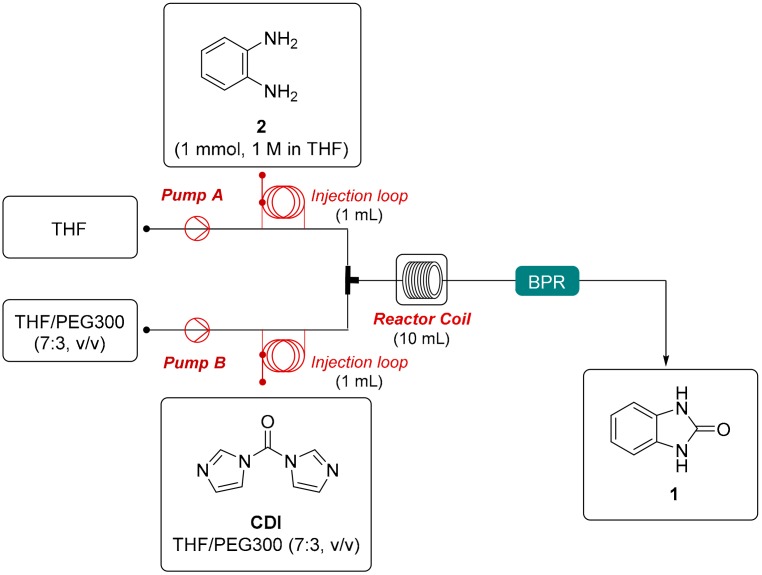
Flow set-up employed for design of experiment (DoE)-assisted optimisation of the model reaction.

Then, we designed a convenient flow set-up for the DoE-assisted optimisation of the model reaction (Figure 2). The reactions were performed by the loop injection of two stock solutions: a solution of *o*-phenylenediamine (**2**, 1 mmol, 1 M) in THF and a solution of CDI in THF/PEG300 (7:3, *v*/*v*). The flow stream was generated by pumping a reservoir of THF and a reservoir of THF/PEG300 (7:3, *v*/*v*), respectively. After the injection and valve switching through the loops, the streams were mixed in a T-piece mixing element and flowed into a 10-mL thermocoupled stainless-steel reactor coil that was heated at the desired temperature. A back-pressure regulator (BPR) (250 psi) was placed following the reactor, thus allowing heating the reaction mixture above its boiling temperature. The output was monitored by a UV detector and readily collected in a fraction collector. The reaction yield was determined by calibrated high-performance liquid chromatography (HPLC) analysis of the crude reaction mixture (Figure 2).

Using this flow set-up, a central composite design (CCD) composed of 14 experiments plus five replicates at the central point was performed to investigate the effect of total flow rate (A), temperature (B), and CDI stoichiometry (C), which are expected to be the main experimental parameters affecting the reaction outcome. In particular, the boundary minimum and maximum values for the selected continuous variables were defined considering the boiling point of the solvent mixture (160 °C) and the equivalent of CDI (2.2) used during the preliminary screening, while the investigated range of flow rates was fixed between 0.10–1.00 mL min^−1^ (which correspond to residence time τ = 100–10 min) in order to maximise the daily productivity (Table 1).

**Figure 3:**
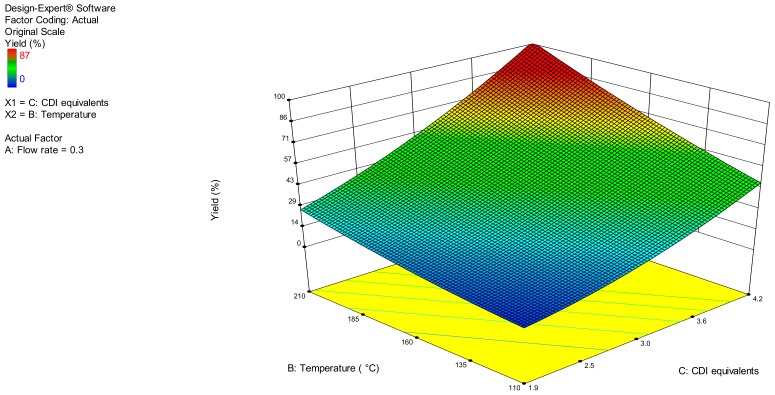
Response surface plot for the optimisation under flow conditions of the model reaction.

Thus, the data acquired (Supplementary Materials and Table 2) were fitted into a linear equation defining a mathematical model (Equation (1)). The analysis of variance (ANOVA) of the model indicated that all the terms were significant (*p*-value < 0.05) (Table 3). Furthermore, the signal-to-noise ratio (adequate precision) and lack of fit were adequate to explore the chemical space, while the statistical and prediction parameters (R^2^, adjusted R^2^ and predicted R^2^) fell within acceptable limits (Table 3). Thus, a three-dimensional response surface was generated according to the mathematical model confirming our hypothesis on the dependence of the measured response (% yield of **1**) on total flow rate (A), temperature (B), and CDI stoichiometry (C) (Figure 3). The DoE software was provided with optimisation criteria based upon the maximisation, minimisation, or evaluation in the explored range of each factor and response, fixing a desirability limit within the optimised conditions. The software furnished different solutions ranked in desirability order, among which the suitable conditions exhibited the highest desirability index. Notably, a high degree of correlation between the predicted and the experimental yield was observed with a total flow rate of 0.3 mL min^−1^ (which corresponds to 33 min of residence time), at a temperature of 210 °C, and in the presence of 4.2 equiv. of CDI, allowing obtaining **1** in 98% yield (Supplementary Materials and Table 4).

Yield^2^ = 4.58 − 1.37A + 1.03B + 2.56C (1)

### 2.2. Gram-Scale Flow Synthesis of N-(2-chlorobenzyl)-5-Cyano-Benzimidazol-2-One (**3**)

**Figure 4:**
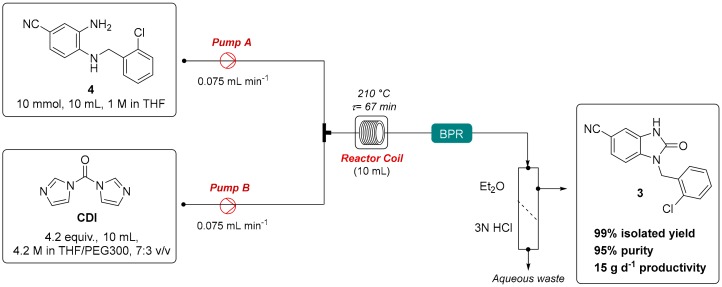
Gram-scale flow synthesis of *N*-(2-chlorobenzyl)-5-cyano-benzimidazol-2-one (**3**).

Thus, the developed optimised protocol was successfully applied for the flow synthesis of *N*-(2-chlorobenzyl)-5-cyano-benzimidazol-2-one (**3**), which is a key synthon for hit-to-lead exploration in anti-inflammatory drug discovery targeting 5-lipoxygenase-activating protein (FLAP) (Figure 4) [26,32]. In this regard, *N*-substituted *o*-phenylenediamine **4**, which was used as the starting material, was synthesised under conventional batch conditions by the reaction of the corresponding *o*-phenylenediamine with 2-chlorobenzyl bromide, as previously reported [22,36]. The flow reaction was performed by continuously pumping a solution of 3-amino-4-((2-chlorobenzyl)amino)benzonitrile (**4**, 2.58 g, 10 mmol, 10 mL, 1 M) in THF and a solution of CDI (6.8 g, 4.2 equiv., 10 mL, 4.2 M) in THF/PEG300 (7:3, *v*/*v*) with a flow rate of 0.075 mL min^−1^ for each pump [37]. The streams were mixed in a T-piece mixing element and flowed into a 10-mL stainless-steel thermocoupled reaction coil (10 mL, τ = 67 min) heated at 210 °C. The reactor output was detected by an in-line UV detector, collected, taken with Et_2_O into a gravity separatory funnel, and washed with 3N HCl to remove PEG300, CDI by-product (imidazole), as well as any trace of unreacted starting material. Then, the collected organic layer was concentrated in vacuo, affording the desired *N*-(2-chlorobenzyl)-5-cyano-benzimidazol-2-one (**3**) intermediate in nearly quantitative isolated yield, high purity (>95%), and with a productivity of 15 g d^−1^ (Supplementary Materials and Figure 4).

## 3. Material and Methods

### 3.1. General Methods

All the chemicals were purchased from Sigma-Aldrich (Milano, Italy) and used without further purification. Melting points were determined using an electrothermal apparatus (B-535, Büchi, Switzerland), and are uncorrected. NMR spectra were recorded on a Bruker AC 400-MHz spectrometer (Bruker, Madison, WI, USA) or on a Varian Mercury 400-MHz spectrometer in the indicated solvent. Chemical shifts are reported in parts per million (ppm) and are relative to *d*^6^-DMSO (2.49 ppm and 39.7 ppm) or CDCl_3_ (7.26 ppm and 77.0 ppm). The abbreviations used are as follows: s, singlet; brs, broad singlet; d, doublet; dd, double of doublets; dt, doublet of triplets; t, triplet; q, quartet; qui, quintet; m, multiplet; and brm, broad multiplet. Coupling constants (*J*) are reported in Hertz (Hz). Thin-layer chromatography (TLC) was performed on aluminum backed silica plates (silica gel 60 F254, Merck, Darmstadt, Germany). Spots were visualised by UV detector (λ: 254 nm) and/or by staining and warming with potassium permanganate. When required, flash chromatographic purifications were performed using Biotage Isolera™ Prime (Biotage AB, Uppsala, Sweden). All the synthesised compounds have been previously reported, and their spectroscopic data were consistent with the literature [21,24,25,35]. All the flow experiments were performed using a Vapourtec R series (Vapourtec Ltd., Bury Saint Edmunds, UK) equipped with two loop injection systems (polytetrafluoroethylene, PTFE, 1 mm ID, 1 mL), two integrated HPLC pumps (R2+ pumping module), a T-piece mixing element (1.5 mm ID), a thermocouple-controlled coil reactor (10 mL, stainless steel, 1.6 mm OD × 1 mm ID) heated at the desired temperature, a back-pressure regulator (BPR, 250 psi, polyether ether ketone, PEEK, 1/16″ OD, 1/4″-28), and a fraction collector (Gilson FC 203B, Gilson, Inc., Middleton, WI, USA). Design of experiments and statistical data analysis was performed using Design-Expert^®^ v. 9 (Stat-Ease, Inc., Minneapolis, MN, USA). Calibrated HPLC analyses were performed on a Shimadzu (Kyoto, Japan) LC-20A Prominence equipped with a CBM-20A communication bus module, two LC-20AD dual piston pumps, a SPD-M20A photodiode array detector, and a Rheodyne 7725i injector (Rheodyne Inc., Cotati, CA, USA) with a 20-μL stainless steel loop. An Ultra II aqueous RP18 column (estek Belle-fonte, PA, USA, 250 mm × 4.6 mm ID, 5 μm, 100 Å) was used as the analytical column. The HPLC analyses were performed using MeOH/H_2_O (25:75, *v*/*v*) + 0.1% diethylamine as the mobile phase at a 1.0 mL min^−1^ eluent flow rate, after previous conditioning by passing through the column the mobile phase for at least 30 minutes at the same eluent velocity. Before use, the mobile phase was filtered through a 0.22-mm Millipore filter (Bedford, MA, USA) and then degassed with 20 minutes of sonication. The column temperature was controlled through a Grace (Sedriano, Italy) heather/chiller (Model 7956 R) thermostat. All the analyses were performed at a 25 °C column temperature. Ultrapure water (ρ = 18.3 MΩ × cm) for HPLC analysis was obtained through New Human machine Power I Scholar (Human Corporation, Seoul, Korea) purification system.

### 3.2. Protocol and Flow Set-Up for DoE Optimisation

The flow setup employed is depicted in Figure 2. A solution of *o*-phenylenediamine (**2**, 108 mg, 1 mmol, 1 M) in THF and a solution of CDI (1.1–5.0 mmol, 1.1–5.0 M) in THF/PEG300 (7:3, *v*/*v*) were injected into the loops and pumped with a flow rate of 0.05–0.5 mL min^−1^ for each pump. After the injection and switching of the valves into the loops, the solutions were mixed together in a T-piece mixing element and flowed through the thermocouple-controlled coil reactor (10 mL, τ = 100–10 min) heated at the desired temperature (110–210 °C). The reactor output was monitored by UV detector and readily collected in a fraction collector. The reaction yield was determined by calibrated HPLC analysis of the crude reaction mixture.

### 3.3. Protocol for the Gram-Scale Flow Synthesis of N-(2-chlorobenzyl)-5-cyano-benzimidazol-2-one (**3**)

The flow set-up employed is depicted in Figure 4. A solution of 3-amino-4-((2-chlorobenzyl)amino)benzonitrile (**4**, 2.58 g, 10 mmol, 10 mL, 1 M) in THF and a solution of CDI (6.8 g, 4.2 equiv., 10 mL, 4.2 M) in THF/PEG300 (7:3, *v*/*v*) were continuously pumped through the HPLC pumps with a flow rate of 0.075 mL min^−1^ for each pump. The streams were mixed in a T-piece mixing element and flowed into a 10-mL stainless-steel thermocoupled reaction coil (10 mL, τ = 67 min) heated at 210 °C. The reactor output was detected by an in-line UV detector, collected, taken with Et_2_O into a gravity separatory funnel, and washed with 3N of HCl to remove any trace of unreacted starting material. Then, the collected organic layer was concentrated in vacuo, affording the desired *N*-(2-chlorobenzyl)-5-cyano-benzimidazol-2-one (**3**) (2.78 g, 9.86 mmol, 99% yield, >95% HPLC purity, 15 g d^−1^ productivity).

### 3.4. Compounds Characterisation

Benzimidazol-2-one (**1**) [36]: White solid (m.p.: > 200 °C, lit. [38]: >250 °C). ^1^H-NMR (400 MHz, *d*^6^-DMSO): δ 6.89 (m, 4H), 10.57 (s, 2H). ^13^C-NMR (100.6 MHz, *d*^6^-DMSO): δ 108.3, 120.3, 129.5, 155.1.

*N*-(2-chlorobenzyl)-5-cyano-benzimidazol-2-one (**3**) [26]: White solid (m.p.: 234–235 °C, lit. [26]: 233.9–234.9 °C). ^1^H-NMR (400 MHz, CDCl_3_): δ 5.24 (s, 2H), 6.93 (d, *J* = 8.2 Hz, 1H), 7.08 (dd, *J*_1_ = 8.0 Hz, *J*_2_ = 1.6 Hz, 1H), 7.17–7.22 (m, 1H), 7.24-7.28 (m, 1H), 7.33 (dd, *J*_1_ = 8.2 Hz, *J*_2_ = 1.4 Hz, 1H), 7.39 (d, *J* = 1.4 Hz, 1H), 7.43 (dd, *J*_1_ = 8.0 Hz, *J*_2_ = 1.6 Hz, 1H), 8.09 (s, 1H). ^13^C-NMR (100.6 MHz, *d*^6^-DMSO): δ 42.0, 103.4, 109.1, 112.3, 120.0, 126.4, 128.0, 128.5, 129.1, 129.7, 130.0, 132.2, 133.8, 134.1, 154.5.

## 4. Conclusions

In this communication, we developed a novel flow method for the preparation of benzimidazol-2-one (**1**) core, which is a well-established privileged scaffold in medicinal chemistry and a common structural framework of numerous biologically active compounds endowed with different therapeutic applications. Thus, starting from a preliminary batch screening, the model cyclocarbonylation reaction of *o*-phenylenediamine (**2**) with CDI was successfully translated under flow conditions and optimised by means of statistical experimental design. In particular, by taking advantage of the multivariate exploration of the reaction chemical space, we screened reaction parameters and analysed their impact on the reaction outcome. In this regard, although our method still requires high temperature, high-boiling solvent, and an excess of CDI to promote the thermal cyclisation–carbonylation reaction, the translation and subsequent optimisation under flow conditions allowed us to obtain high reproducibility especially on larger scale, as demonstrated by the multigram-scale continuous synthesis of *N*-(2-chlorobenzyl)-5-cyano-benzimidazol-2-one (**3**), a key intermediate for the preparation of FLAP inhibitors [32]. Future efforts will be directed towards the implementation of this flow platform by automation for the preparation of *N*-substituted and *N*,*N*′-disubstituted benzimidazolone-based compound library, which will be useful for hit-to-lead exploration in our anti-inflammatory drug discovery program.

## Figures and Tables

**Table 1:** Variable settings for central composite design (CCD).

Variable Name	Variable Units	Range
Total flow rate (A)	mL min^−1^	0.10–1.00
Temperature (B)	°C	110–210
CDI stoichiometry (C)	equivalents	1.1–5.0

**Table 2:** CCD: experimental matrix and measured responses. *^a^*

Run	Type	Factor A (mL min^−1^)	Factor B (°C)	Factor C (equiv.)	Yield *^b^*
1	Factorial	0.3	190	1.9	10
2	Factorial	0.8	190	1.9	3
3	Axial	1.0	160	3.0	8
4	Center	0.6	160	3.0	32
5	Center	0.6	160	3.0	27
6	Axial	0.6	160	1.1	0
7	Center	0.6	160	3.0	27
8	Factorial	0.3	130	1.9	0
9	Factorial	0.8	190	4.2	61
10	Factorial	0.8	130	4.2	13
11	Factorial	0.3	130	4.2	78
12	Axial	0.6	110	3.0	6
13	Factorial	0.8	130	1.9	0
14	Factorial	0.3	190	4.2	84
15	Center	0.6	160	3.0	32
16	Axial	0.1	160	3.0	87
17	Axial	0.6	210	3.0	38
18	Axial	0.6	160	5.0	38
19	Center	0.6	160	3.0	25

*^a^* Reactions were conducted according to Figure 2 using 1 mmol of **2**. *^b^* Determined by calibrated HPLC analysis.

**Table 3:** ANOVA results and associated statistics related to the response surface model.

Source	Sum of Squares	Mean Square	F Value	*p*-Value Prob > F *^a^*	β_i_ *^b^*	Std. error	95% CI Low	95% CI High
Model	129.41	43.14	22.60	<0.0001 *signif.*	-			
Intercept	-				4.58	0.32	3.90	5.25
A	25.57	25.57	13.40	0.0023	−1.37	0.37	−2.17	−0.57
B	14.42	14.42	7.56	0.0149	1.03	0.37	0.23	1.82
C	89.42	89.42	46.86	<0.0001	2.56	0.37	1.76	3.36
Lack of Fit *^c^*	26.23	2.38	3.98	0.0969 *non-signif.*	-			
Std. Dev. = 1.38 Mean = 4.58 C.V. % = 30.17 PRESS = 50.88					R^2^ = 0.9827 Adj. R^2^ = 0.9791 *^d^* Pred. R^2^ = 0.9602 *^d^* Adeq. Prec. = 15.634 *^e^*			

*^a^p*-value “Prob > F” minor than 0.0500 indicates model terms are significant. *^b^* Estimated regression codes coefficients variable. *^c^* Non-significant lack of fit means the model fits. *^d^* The difference between adjusted R^2^ and predicted R^2^ has to be minor than 0.2. *^e^* Adeq. Prec. measures the signal to noise ratio: a ratio greater than four is desirable so that the model can be used to navigate the chemical space.

**Table 4:** Predicted and experimental results for the selected optimisation criteria. *^a^*

Optimisation Criteria	A (mL min^−1^)	B (°C)	C (CDI equiv.)	Predicted Yield (%)	Experimental Yield (%) *^b^*
Maximise X A, B, and C in range	0.3	210	4.2	99	98

*^a^* Reactions were conducted according to Figure 2 using 1 mmol of **2**. *^b^* Determined by calibrated HPLC analysis.

## Supplementary Materials

The following are available online. Figures S1–S4: ^1^H-NMR and ^13^C-NMR spectra of compounds **1** and **3**; Figures S5 and S6: HPLC calibration curves for compounds **1** and **2**; Figure S7: Overview of the results based on calibration curves; Figures S8 and S9: HPLC chromatograms of compounds **1** and **2** reference standard; Figures S10 and S11: HPLC chromatograms of crude compounds **1** and **3** obtained under optimised flow conditions.
