# Supplementary material for: Optimisation by Design of Experiment of Benzimidazol-2-One Synthesis under Flow Conditions
Source: Molecules. 2019 Jul 3;24(13):2447. doi: 10.3390/molecules24132447 (PMC6651037; doi:10.3390/molecules24132447)
Supplement: Supplementary file 1 [file molecules-24-02447-s001.pdf]

SUPPORTING INFORMATION

Optimisation by Design of Experiment of Benzimidazol-2-One Synthesis under Flow Conditions

Serena Mostarda [1,2,†], Tugçe Gür Maz [3,†], Alessandro Piccinno [1] Bruno Cerra [1,*] and Erden Banoglu [3,*]

[1] Department of Pharmaceutical Sciences, University of Perugia, Via del Liceo 1, 06123, Perugia, Italy; serena.mostarda@novartis.com (S.M.); alessandro.piccinno@chimfarm.unipg.it (A.P.)
[2] Current affiliation: Novartis Pharma AG, CH-4002 Basel, Switzerland
[3] Department of Pharmaceutical Chemistry, Faculty of Pharmacy, Gazi University, Etiler, 06560, Ankara, Turkey; ztugcegur@gmail.com (T.G.M.)
[*] Correspondence: bruno.cerra@chimfarm.unipg.it (B.C.); banoglu@gazi.edu.tr (E.B.); Tel.: +39-075-5855120 (B.C.) Tel.: +90-31-2023236 (E.B.)
[†] These authors contributed equally to this work.

Figure S1: ^{1}H-NMR of compound 1 (d^6-DMSO, 400 MHz)

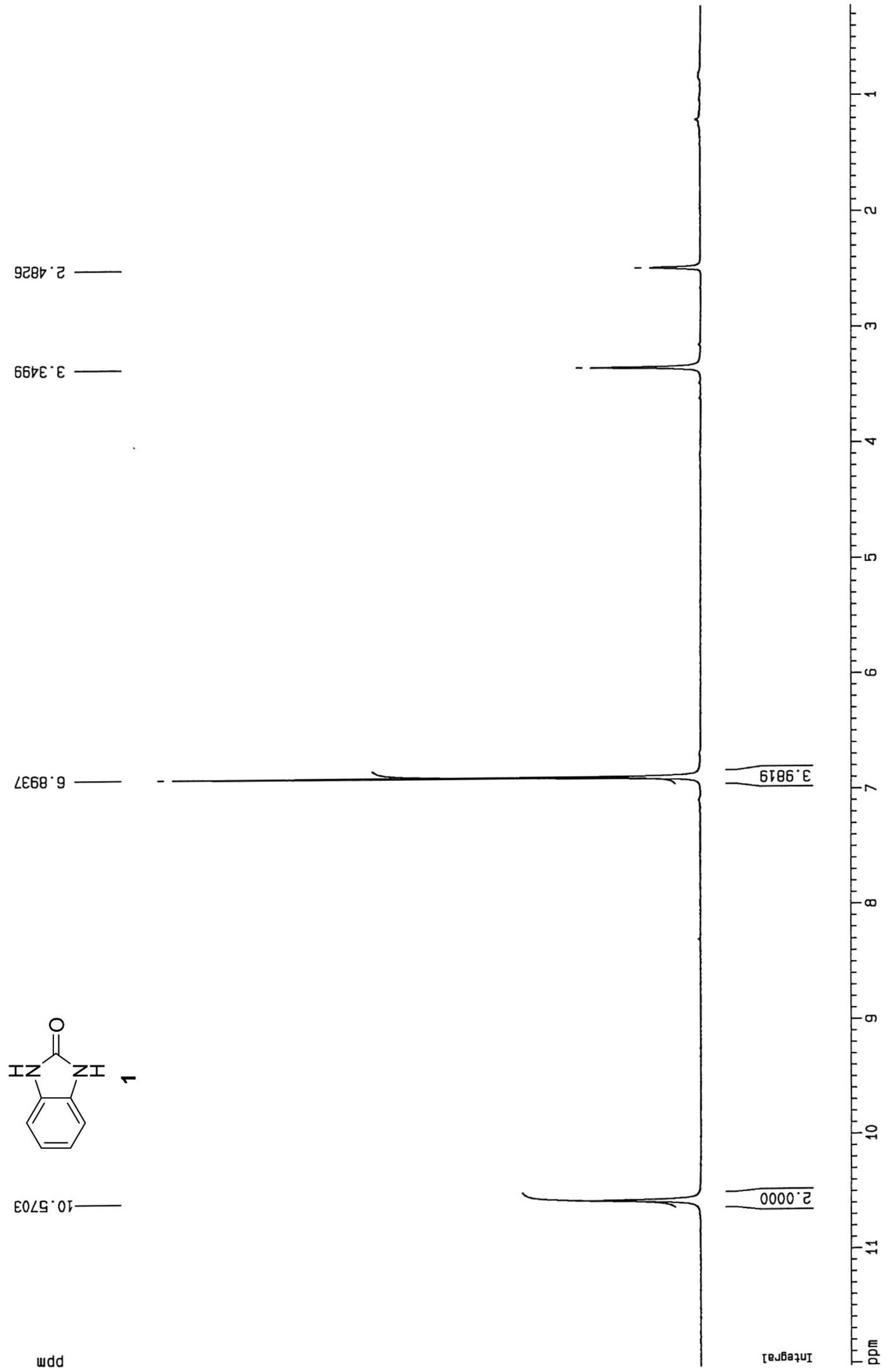

Figure S2: ^{13}C-NMR of compound 1 (d^6-DMSO, 100.6 MHz)

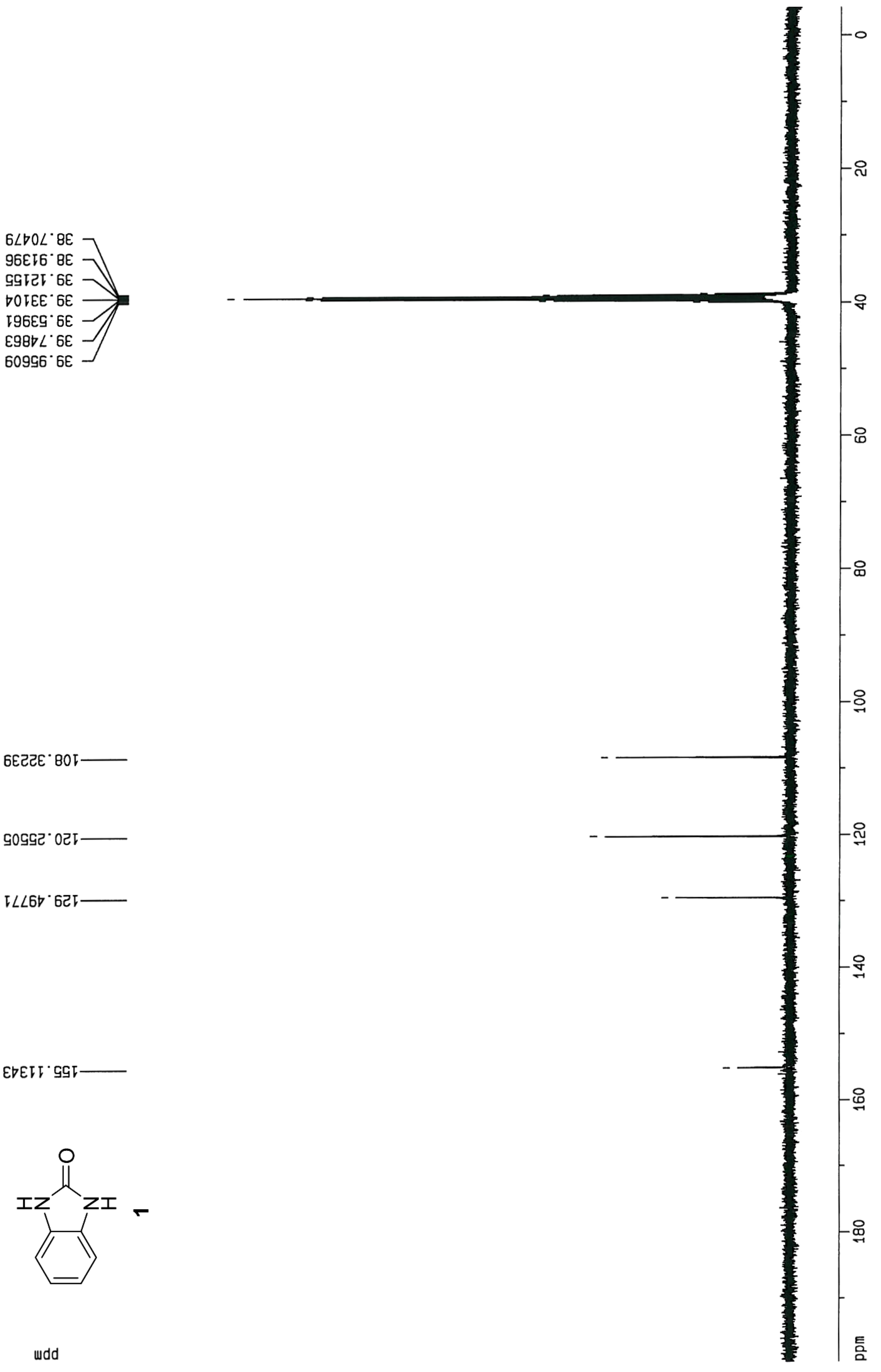

Figure S3: ^{1}H-NMR of compound 3 ($CDCl_3$, 400 MHz)

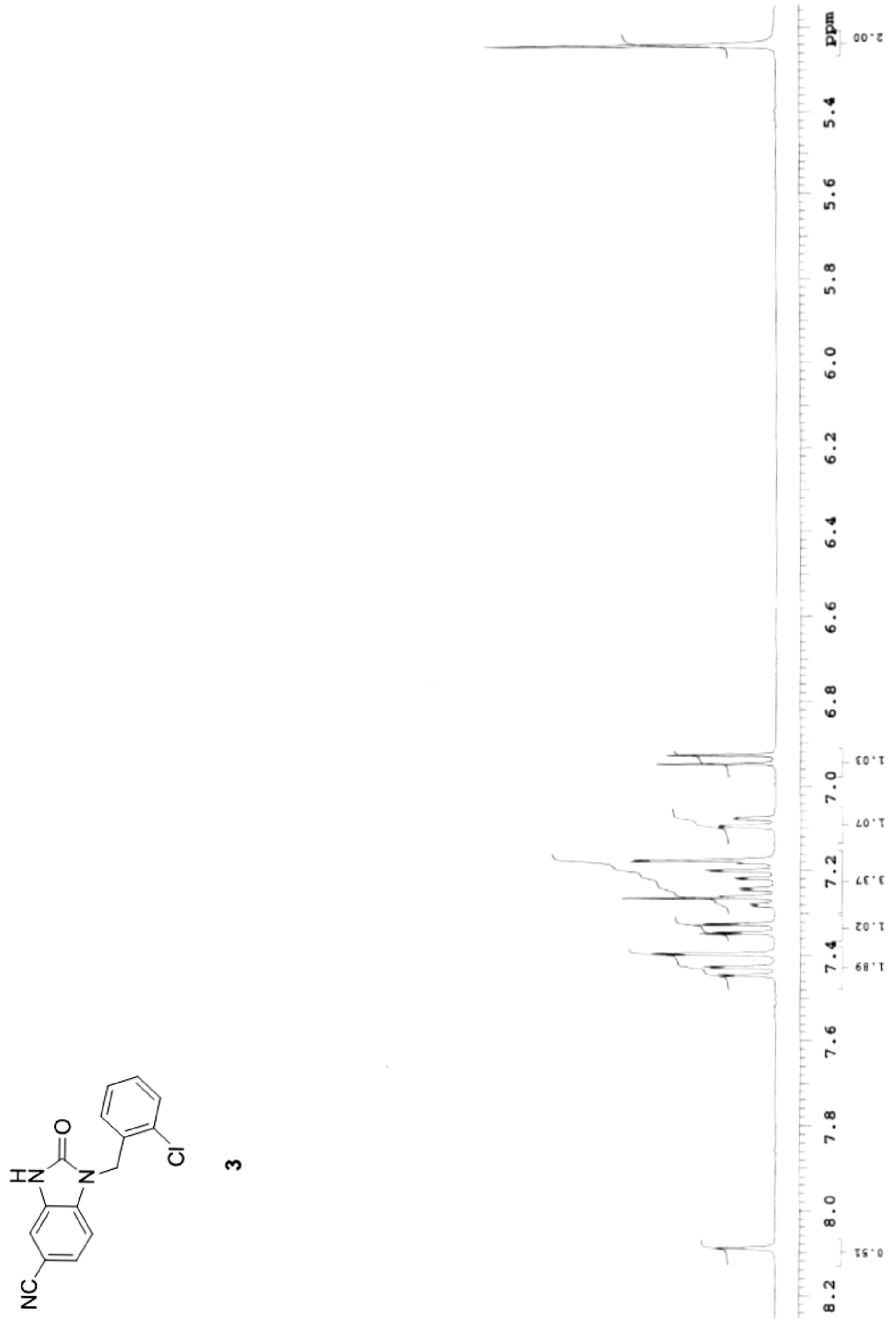

Figure S4: ^{13}C-NMR of compound 3 (d^6-DMSO, 100.6 MHz)

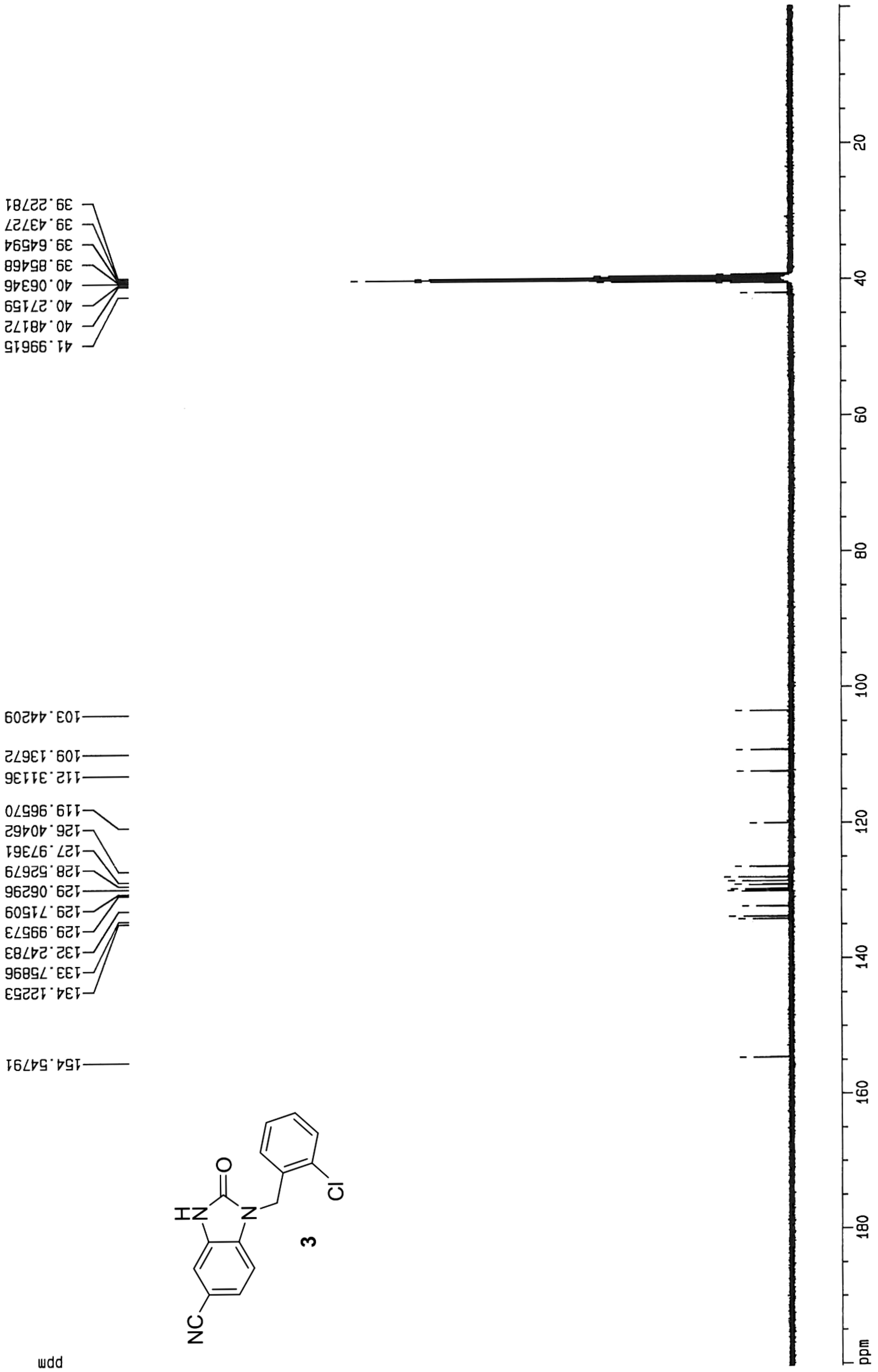

Figure S5: HPLC calibration curve for compound 1

Conc (mM)	Area
0,06	43726,808
0,036	30313,024
0,018	16817,372
0,001	821,07
0,0001	134,443

Slope	7,41E+05
Intercept	1,31E+03
R^2	0,9939

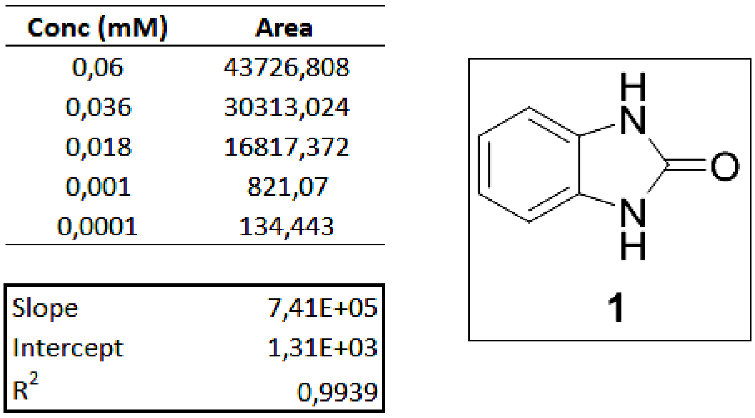

1

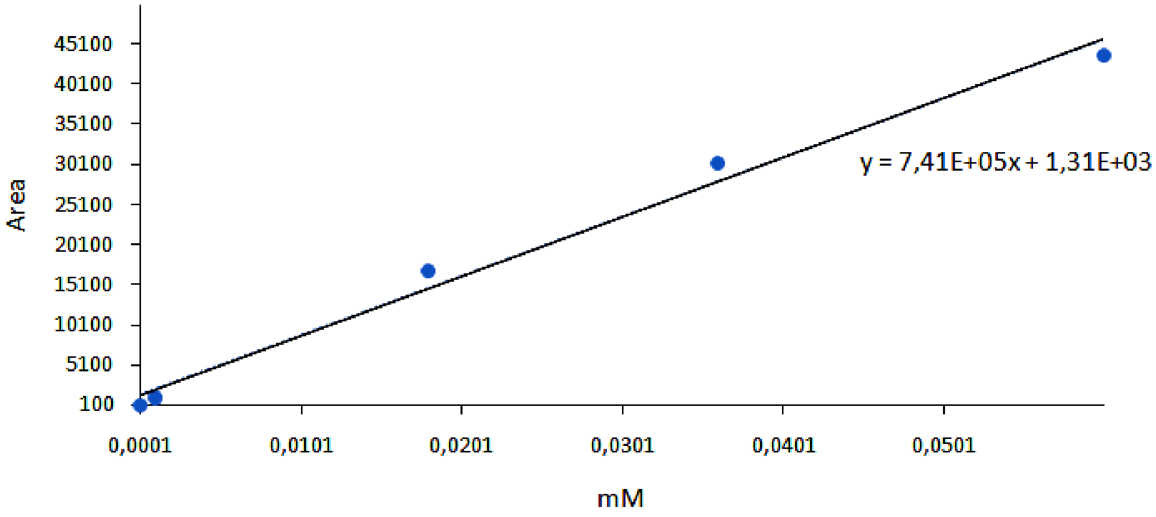

Figure S6: HPLC calibration curve for compound 2

Conc (mM)	Area
0,019	29482,197
0,018	28993,424
0,016	25437,047
0,015	23108,462
0,013	21761,275

Slope	1,42E+06
Intercept	2,82E+03
R^2	0,9817

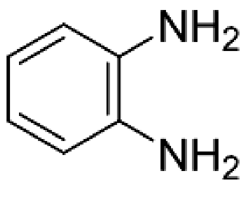

2

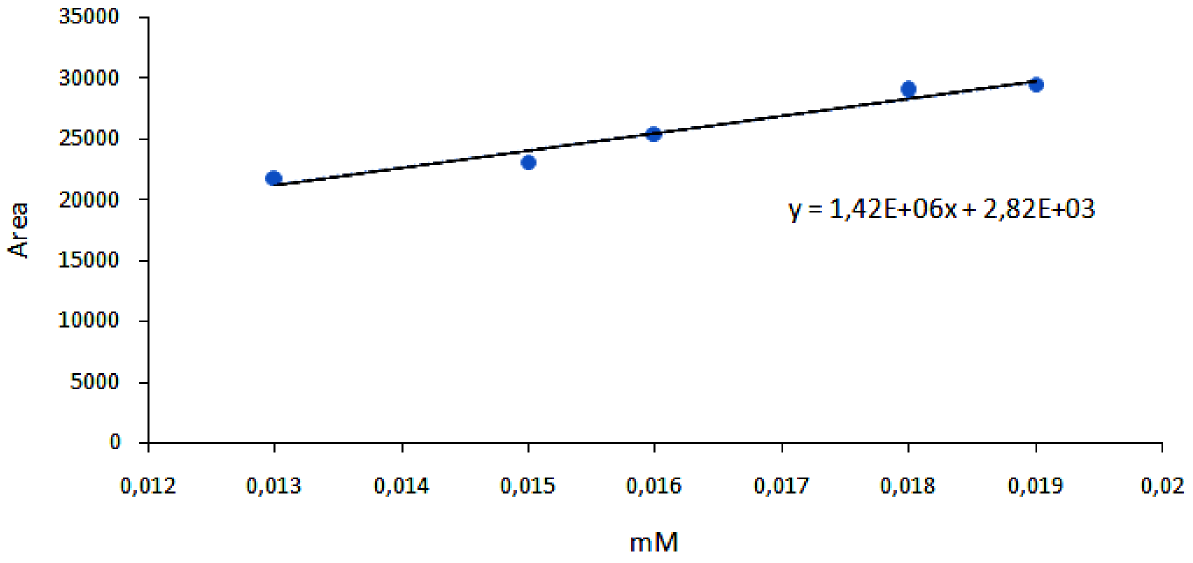

Figure S7: Overview of the results based on calibration curves

	Slope	1415994			Slope	740799	
	Intercept	2817			Intercept	1309	
	R^2	0,9817			R^2	0,9939	
	o-Phenylenediamine (2)				Benzimidazolone (1)		
	Area	mM	%		Area	mM	%
Run 1 DoE Table 3	29969	0,01917	89,7		2939	0,002200	10,3
Run 2 DoE Table 3	35533	0,02310	96,8		1880	0,000771	3,2
Run 3 DoE Table 3	27092	0,01714	91,7		2461	0,001555	8,3
Run 4 DoE Table 3	27863	0,01769	67,8		7537	0,008407	32,2
Run 5 DoE Table 3	26964	0,01705	72,6		6085	0,006447	27,4
Run 6 DoE Table 3	33891	0,02194	100,0		57	0,000000	0,0
Run 7 DoE Table 3	19388	0,01170	73,0		4513	0,004325	27,0
Run 8 DoE Table 3	36555	0,02383	100,0		183	0,000000	0,0
Run 9 DoE Table 3	41151	0,02707	39,3		32272	0,041796	60,7
Run 10 DoE Table 3	25194	0,01580	87,4		2993	0,002273	12,6
Run 11 DoE Table 3	8126	0,00375	22,3		10974	0,013046	77,7
Run 12 DoE Table 3	33615	0,02175	93,6		2403	0,001476	6,4
Run 13 DoE Table 3	37313	0,02436	100,0		11	0,000000	0,0
Run 14 DoE Table 3	6551	0,00264	16,2		11447	0,013685	83,8
Run 15 DoE Table 3	19021	0,01144	67,7		5345	0,005448	32,3
Run 16 DoE Table 3	8918	0,00431	13,4		21881	0,027769	86,6
Run 17 DoE Table 3	18506	0,01108	61,9		6369	0,006829	38,1
Run 18 DoE Table 3	18330	0,01096	61,7		6340	0,006790	38,3
Run 19 DoE Table 3	19108	0,01150	74,6		4217	0,003926	25,4
Check point Table 5	3402	0,00041	2,0		16284	0,020214	98,0

Figure S8: HPLC chromatogram of compound 1 (reference standard)

SAMPLE: Benzimidazol-2-one0.018M
:
Vial number: 4
Volume: 20.0 µl
Dilution: 1.00
Amount: 1.0000

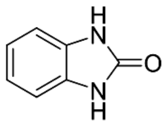

1

COLUMN: Ultra II Aqueous
Size: 4.6 x 250 mm
Number:
Part.size: 5.0 µm

ELUENT: MeOH/H2O-25/75 (v,v) + DEA 0.1%

Flow: 0.00 mL/min
Temperature: -273.0°C
Pressure: -145.0 psi

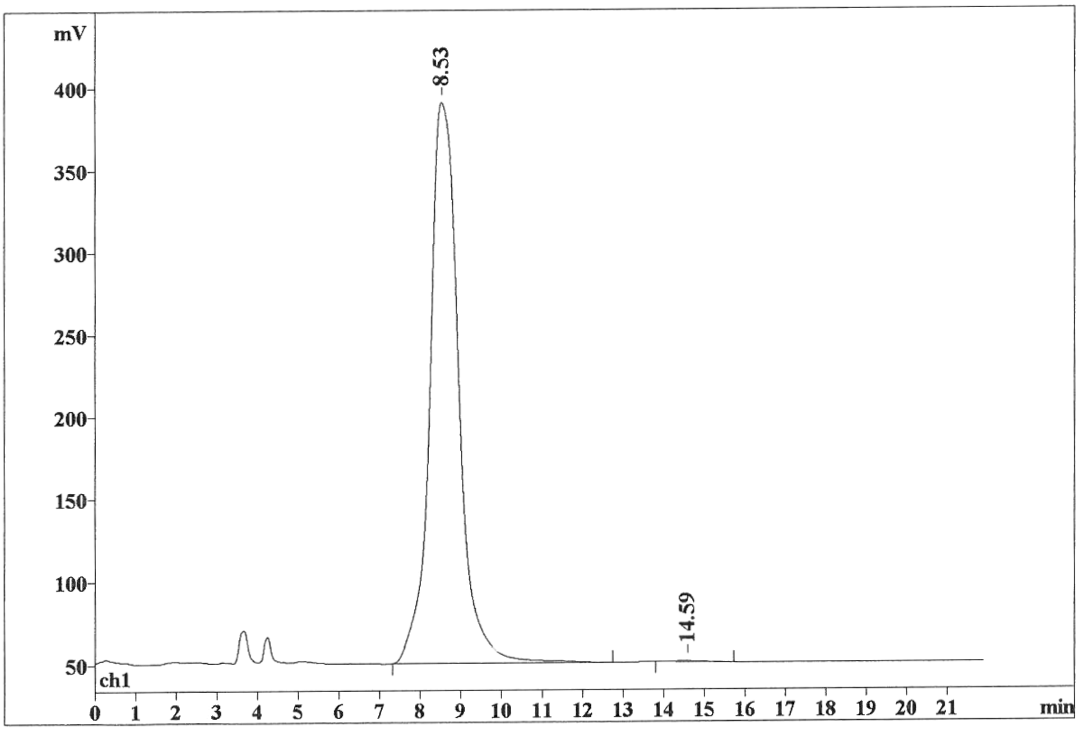

Quantitation method: Custom

Retention min	Width/2 min	Height mV	Height %	Area mV*sec	Area %
8.53	0.708	340.48	99.84	16817.372	99.83

Figure S9: HPLC chromatogram of compound 2 (reference standard)

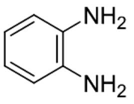

2

SAMPLE:	Diamine0.015M
:	
Vial number:	4
Volume:	20.0 µl
Dilution:	1.00
Amount:	1.0000
COLUMN:	Ultra II Aqueous
Size:	4.6 x 250 mm
Number:	
Part.size:	5.0 µm
ELUENT:	MeOH/H2O-25/75 (v,v) + DEA 0.1%
Flow:	0.00 mL/min
Temperature:	-273.0°C
Pressure:	-145.0 psi

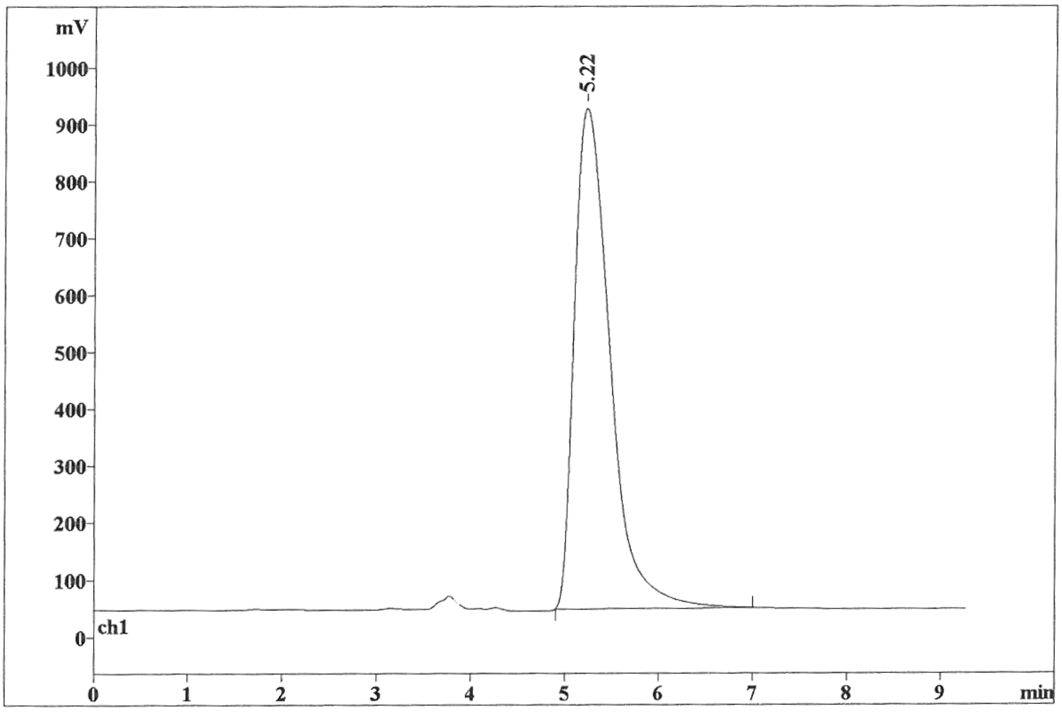

Quantitation method: Custom

Retention min	Width/2 min	Height mV	Height %	Area mV*sec	Area %
5.22	0.399	878.83	100.00	23108.462	100.00

Figure S10: HPLC chromatogram of crude 1 obtained by optimised flow conditions (Table 5)

Vial number: 4
Volume: 20.0 µl
Dilution: 1.00
Amount: 1.0000

COLUMN: Ultra II Aqueous
Size: 4.6 x 250 mm
Number:
Part.size: 5.0 µm

ELUENT: MeOH/H2O-25/75 (v,v) + DEA 0.1%

Flow: 0.00 mL/min
Temperature: -273.0°C
Pressure: -145.0 psi

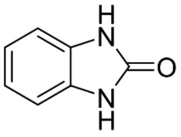

1

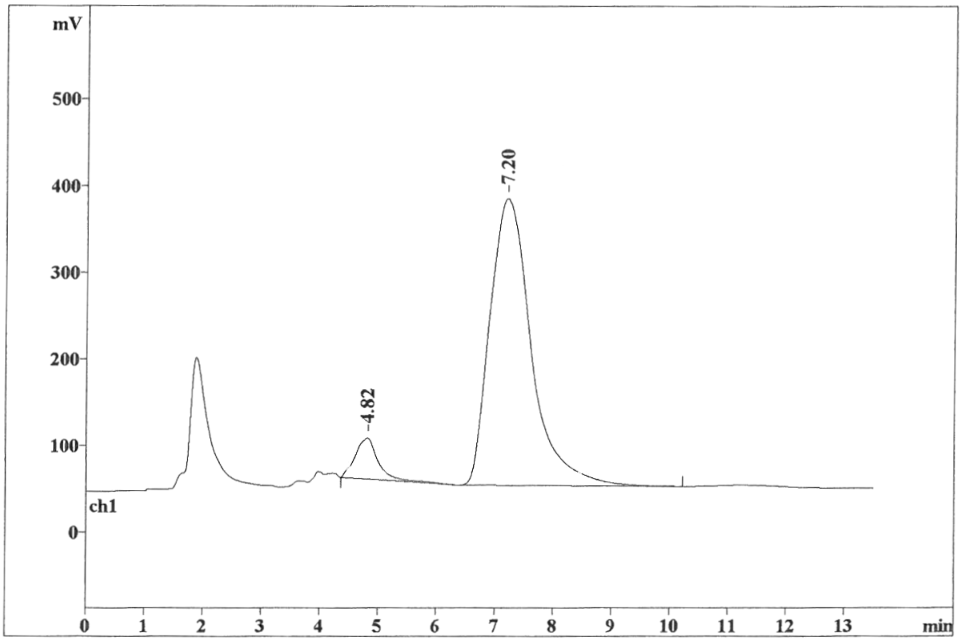

Quantitation method: Custom

Retention min	Width/2 min	Height mV	Height %	Area mV*sec	Area %	k
4.82	0.387	47.22	12.48	1312.609	7.46	0.00
7.20	0.734	331.05	87.52	16284.211	92.54	0.00
13.51	0.560	378.27	100.00	17596.821	100.00	0.00

Figure S11: HPLC chromatogram of crude 3 obtained by optimised flow conditions (Figure 4)

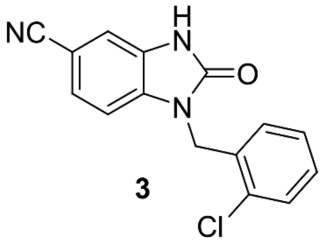

3

Volume:	20.0 µl
Dilution:	1.00
Amount:	1.0000
COLUMN:	Ultra II Aqueous
Size:	4.6 x 250 mm
Number:	
Part.size:	5.0 µm
ELUENT:	MeOH/H2O-25/75 (v,v) + DEA 0.1%
Flow:	0.00 mL/min
Temperature:	-273.0°C
Pressure:	-145.0 psi

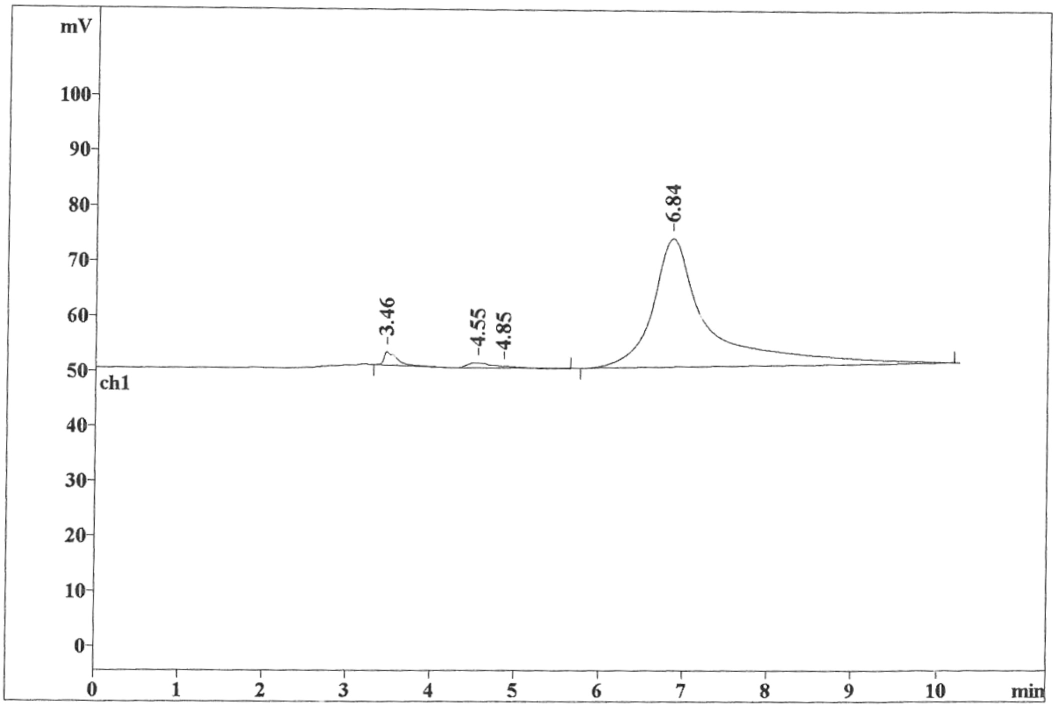

Quantitation method: Custom

Retention min	Width/2 min	Height mV	Height %	Area mV*sec	Area %	k
3.46	0.158	2.48	9.09	28.257	2.45	0.00
4.53	0.352	0.94	3.45	19.044	1.65	0.00
4.85	0.148	0.41	1.49	5.298	0.46	0.00
6.84	0.492	23.45	85.98	1100.167	95.44	0.00
